# CrackNet-Weather: An Effective Pavement Crack Detection Method Under Adverse Weather Conditions

**DOI:** 10.3390/s25175587

**Published:** 2025-09-07

**Authors:** Wei Wang, Xiaoru Yu, Bin Jing, Ziqi Tang, Wei Zhang, Shengyu Wang, Yao Xiao, Shu Li, Liping Yang

**Affiliations:** 1 College of Computer Science and Technology, Changchun University, No. 6543, Satellite Road, Changchun 130022, China; 2 School of Construction Engineering, Jilin University, No. 2699, Qianjin Street, Changchun 130012, China

**Keywords:** pavement crack detection, CrackNet-Weather, Haar Wavelet Downsampling Block, Strip Pooling Bottleneck Block, Dynamic Sampling Upsampling Block

## Abstract

Accurate pavement crack detection under adverse weather conditions is essential for road safety and effective pavement maintenance. However, factors such as reduced visibility, background noise, and irregular crack morphology make this task particularly challenging in real-world environments. To address these challenges, we propose CrackNet-Weather, which is a robust and efficient detection method that systematically incorporates three key modules: a Haar Wavelet Downsampling Block (HWDB) for enhanced frequency information preservation, a Strip Pooling Bottleneck Block (SPBB) for multi-scale and context-aware feature fusion, and a Dynamic Sampling Upsampling Block (DSUB) for content-adaptive spatial feature reconstruction. Extensive experiments conducted on a challenging dataset containing both rainy and snowy weather demonstrate that CrackNet-Weather significantly outperforms mainstream baseline models, achieving notable improvements in mean Average Precision, especially for low-contrast, fine, and irregular cracks. Furthermore, our method maintains a favorable balance between detection accuracy and computational complexity, making it well suited for practical road inspection and large-scale deployment. These results confirm the effectiveness and practicality of CrackNet-Weather in addressing the challenges of real-world pavement crack detection under adverse weather conditions.

## 1. Introduction

Pavement crack detection is essential for traffic safety and road infrastructure maintenance. With more than 64 million kilometers of roads worldwide [1], pavement defects such as cracks and potholes are major contributors to vehicle damage and traffic accidents [2]. According to the World Health Organization, road traffic crashes claim over 1.3 million lives each year, and adverse weather conditions—including rain and snow—can increase accident rates by up to 50% [3]. These extreme weather events not only accelerate pavement deterioration but also significantly complicate crack detection, as visibility is reduced and road surfaces become obscured by precipitation [4]. In such environments, rain streaks and snowflakes act as visual noise, masking surface textures and interfering with conventional image-based detection algorithms [5]. Consequently, existing detection systems often struggle to reliably recognize cracks under adverse weather conditions [6]. This underscores the urgent need for robust and adaptive crack detection methods that can maintain high accuracy and stability across a wide range of challenging weather scenarios.

Conventional approaches for identifying pavement cracks—such as manual surveys or basic image analysis—have long been the norm, but these methods are inefficient and easily influenced by human subjectivity and environmental changes [7,8]. As technology has advanced, deep learning and computer vision have transformed the field with models like YOLO [9,10,11,12,13,14,15,16] becoming popular for their speed and accuracy in detecting pavement damage. These models excel at handling large datasets and learning diverse visual patterns without handcrafted features [17]. Despite their strengths, most current models are developed and validated using datasets captured in good weather [18], leaving them vulnerable when applied to images from rainy or snowy conditions. Rain, snow, and lighting variations introduce complex visual disturbances, further reducing the reliability of crack detection in real-world scenarios [19]. This gap underlines the need for robust detection solutions that remain effective regardless of the weather [20].

Object detection constitutes a cornerstone of computer vision research and continues to evolve rapidly, which is propelled by the growing demand for high-precision and real-time automated inspection systems [21,22]. The YOLO (You Only Look Once) family of detectors [23] has played a pivotal role in this evolution. Meanwhile, methods like EfficientDet [24] have introduced compound scaling and attention-based modules to optimize detection efficiency, and transformer-based [25] frameworks such as DETR [26] have redefined detection paradigms through end-to-end architectures. In the specific context of pavement crack detection, these object detection models enable the rapid, accurate localization of surface defects across varied and challenging environments. Compared to segmentation-based approaches [27], detection models provide superior inference speed and adaptability, greatly improving the practicality and reliability of large-scale road monitoring systems. Collectively, these methodological advances establish a strong technological foundation for robust crack detection, especially in scenarios requiring resilience to complex weather-induced visual disturbances [28]. However, even with these advances, there remains a need for further architectural improvements to address the unique challenges posed by extreme weather in pavement crack detection.

**Figure 1:**
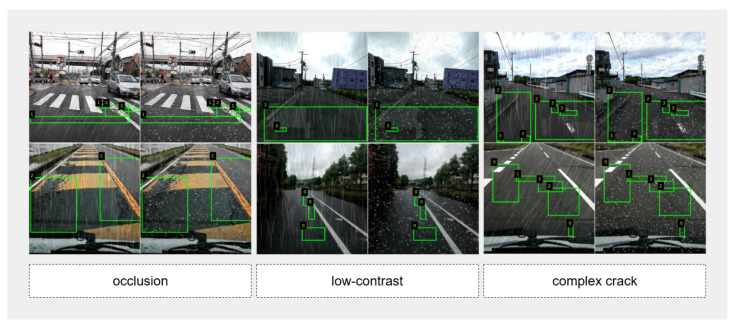
Representative challenging cases in pavement crack detection under adverse weather conditions. (**Left**) **Occlusion**: Environmental objects partially obscure crack regions. (**Middle**) **Low-contrast**: Rain or snow reduces the visibility of cracks against the pavement background. (**Right**) **Complex crack**: Irregular and fragmented crack patterns make detection and localization more difficult. The green bounding boxes indicate annotated defect regions.

The pavement crack detection task involves identifying several common types of pavement distress. In this study, the dataset [29] is divided into four defect categories: class_0 (longitudinal cracks), class_1 (transverse cracks), class_2 (alligator cracks), and class_3 (potholes), which represent the most prevalent and safety-critical forms of road surface damage encountered in real-world scenarios. In practical road monitoring applications, extreme weather presents several key challenges to accurate crack detection, as illustrated in Figure 1. These challenges are summarized as follows: Low visibility refers to situations where rain, fog, or other adverse weather conditions blur or darken the scene, reducing the clarity and visibility of cracks. Low visibility often leads to missing or incomplete crack region detection, particularly for fine or shallow cracks [30,31,32]. Background noise is characterized by the presence of extraneous visual elements such as raindrops, snowflakes, shadows, vehicles, or strong road markings. These background elements act as visual occluders, masking or confusing the true crack features, and they can result in false detections or missed cracks. Complex cracks refers to defects that are irregular, fragmented, or highly interconnected—such as alligator cracks. Their irregular shapes and spatial distribution increase the difficulty of both detection and accurate localization, especially under adverse environmental conditions. Figure 1 provides representative examples of these challenging scenarios. The first group of images demonstrates occlusion and background noise caused by precipitation and other environmental factors; the second group illustrates the reduced contrast and blurred features in low-visibility conditions; and the third group shows the challenges associated with complex and fragmented crack patterns. The green bounding boxes in each image indicate the annotated defect regions. These challenges highlight the urgent need for robust pavement crack detection frameworks capable of addressing environmental interference and preserving detection accuracy in real-world, weather-perturbed environments.

To address the aforementioned challenges, this paper proposes an improved pavement crack detection method based on YOLOv12. The method systematically incorporates three key modules aimed at enhancing feature robustness, spatial detail preservation, and adaptability in adverse weather conditions. Specifically, we replace the standard downsampling convolutions in the backbone with the Haar Wavelet Downsampling Block (HWDB) [33], which effectively preserves both low-frequency and high-frequency information during spatial reduction. In both the backbone and neck, all Cross-Stage Partial modules with 2 × 2 convolutional kernels (C3K2) are replaced by the Strip Pooling Bottleneck Block (SPBB) [34], which achieves multi-scale and directional context aggregation and improves the detection of elongated, fragmented, and low-contrast cracks. In the upsampling stage, the Dynamic Sampling Upsampling Block (DSUB) [35] is introduced in place of conventional upsampling operators, enabling content-adaptive spatial feature reconstruction and enhanced boundary detail recovery.

The main contributions of this paper are summarized as follows:

(1) To address the scarcity of extreme weather samples, we selected 2600 images from the RDD2022 [29] Japan dataset and applied simulation algorithms to generate realistic rain and snow conditions for each image. This process resulted in a diverse experimental dataset encompassing clear, rainy, and snowy scenarios, which provides a solid data foundation for robust crack detection research in complex and adverse environments.

(2) We propose a novel pavement crack detection method based on YOLOv12, integrating the HWDB, SPBB, and DSUB modules, which significantly enhances detection performance under complex and adverse weather conditions.

(3) To address the challenges of information loss and poor recognition of low-contrast cracks caused by precipitation and occlusion, we design the HWDB module, which effectively preserves detailed and edge information through Haar wavelet decomposition.

(4) To tackle the problems of elongated, fragmented, and directionally ambiguous cracks, the SPBB module is introduced in both the backbone and neck, leveraging strip pooling and multi-branch context aggregation to enhance the representation of directional and global features.

(5) To improve the recovery of fine details and crack boundaries in the upsampling stage, we construct the DSUB module, which enables a content-aware reconstruction of high-resolution feature maps via the adaptive prediction of spatial offsets and scaling.

(6) Extensive experiments on the aforementioned multi-scenario datasets as well as the Norwegian dataset of RDD2022 show that our approach achieves superior accuracy and robustness compared to mainstream baseline methods, especially under severe weather conditions.

## 2. Related Work

### 2.1. Recent Progress in Object Detection

Modern object detection has been revolutionized by the rapid evolution of one-stage detectors, among which the YOLO (You Only Look Once) family stands out as the most prominent and widely adopted framework. Classic versions such as YOLOv5 and YOLOv8 have set benchmarks for balancing accuracy and inference speed, while the release of YOLOv10 [13], YOLOv11 [14],YOLOv12 [15] and the latest YOLOv13 [16] have further advanced the state of the art through innovations in network architecture, training strategies, and model scalability. In addition, transformer-based detectors such as DETR [26] and RT-DETR [36] have introduced new paradigms for end-to-end object detection, leveraging global attention mechanisms to improve robustness in complex visual environments. These models have been increasingly applied to road surface inspection tasks, enabling the efficient and accurate identification of various pavement defects, including longitudinal cracks, transverse cracks, alligator cracks, and potholes. In parallel, several effective architectural modules have been introduced to further enhance the performance and robustness of object detection models. For instance, Xu et al. [33] designed a Haar wavelet-based downsampling module that better preserves spatial and boundary information, improving segmentation accuracy. Hou et al. [34] proposed strip pooling, which is a lightweight spatial pooling method that efficiently captures long-range contextual information for scene parsing. Liu et al. [35] presented DySample, which is an ultra-lightweight and efficient dynamic upsampling module based on content-aware point sampling, which achieves superior accuracy and computational efficiency across various visual tasks. Nevertheless, even with these advanced detectors, substantial challenges remain when operating under extreme weather conditions such as heavy rain or snow [37]. Adverse environments introduce occlusions, visual artifacts, and reduced contrast, which hinder reliable feature extraction and increase the rates of false positives and missed detections [38]. These limitations highlight the pressing need for enhanced detection frameworks that can maintain robustness and accuracy in real-world, weather-perturbed scenarios.

### 2.2. Similar Works

Many researchers have already undertaken similar work on solving the problem of pavement crack detection [39,40,41]. Gavilan et al. [42] propose an adaptive pavement crack detection framework that leverages pavement classification and multi-stage preprocessing to improve the reliability of automatic road distress assessment. The system utilizes an SVM-based classifier ensemble to tailor detection parameters to different pavement types, effectively reducing false positives from non-crack features and enhancing overall crack detection accuracy. Hu et al. [43] employ YOLOv5-based deep learning models for automatic pavement crack detection, demonstrating superior accuracy and speed over traditional approaches. Their work highlights the effectiveness of deep learning for identifying cracks of varying severity, though challenges persist in more complex or visually degraded environments. Fan et al. [44] propose a shadow-removal-oriented crack detection framework and introduce a dedicated shadow-crack dataset to address the interference of shadows in pavement crack detection. Their method effectively improves detection accuracy in the presence of shadows and varying brightness caused by seasonal and weather changes. Despite significant progress in deep learning-based pavement crack detection, robust and efficient detection under adverse weather conditions remains an open challenge. Existing models often experience degraded performance when dealing with low visibility, background noise, and complex crack morphologies. Motivated by these gaps, this paper presents CrackNet-Weather, a model that systematically enhances frequency information preservation, context-aware feature fusion, and content-adaptive upsampling, aiming to achieve robust pavement crack detection in rain, snow, and other challenging weather scenarios.

## 3. Methodology

### 3.1. Architecture of the Proposed Method

**Figure 2:**
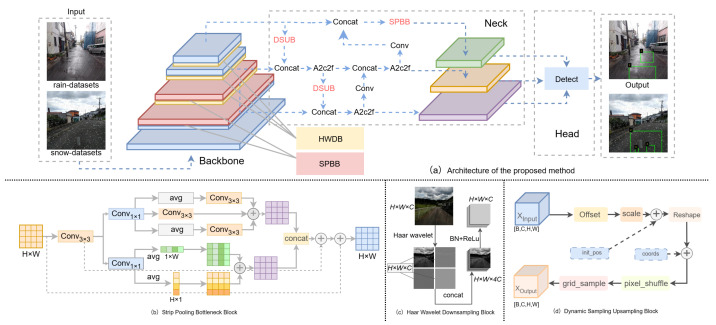
Architecture of the proposed method.

As illustrated in Figure 2a, we propose a substantially enhanced pavement crack detection framework, built upon YOLOv12 [15], that is specifically tailored for the challenging conditions encountered in adverse weather scenarios. Recognizing that standard detection architectures often fail to maintain accuracy in the presence of reduced visibility, precipitation-induced noise, and irregular crack morphologies, we systematically redesign the backbone and upsampling stages of YOLOv12. By introducing specialized modules at these key locations, our approach significantly improves the network’s robustness and detection performance under real-world rainy and snowy environments.

In the backbone, we replace all standard convolutional downsampling layers with the Haar Wavelet Downsampling Block (HWDB) [33]. Conventional downsampling operations, such as strided convolution or pooling, frequently result in the loss of crucial edge and texture details—a limitation that becomes even more severe in the presence of low-contrast or partially occluded regions caused by adverse weather. To address this challenge, the HWDB leverages Haar wavelet transformation to decompose feature maps into both low- and high-frequency components, enabling the network to capture subtle crack boundaries and fine structural cues that would otherwise be eliminated by traditional methods. This targeted integration markedly enhances the network’s capacity to preserve and propagate essential features, leading to the more reliable detection of faint, blurred, or partially obscured cracks in complex real-world conditions.

For multi-scale and contextual feature fusion, we replace all C3K2 modules with the Strip Pooling Bottleneck Block (SPBB) [34]. While the original C3K2 modules are computationally efficient, they exhibit clear limitations in capturing long-range dependencies and directional characteristics—an ability that is critical for accurately detecting fragmented or elongated cracks, particularly when these cracks are partially obscured by precipitation such as raindrops or snowflakes. By adopting a dual-branch design that incorporates both strip pooling and global average pooling, the SPBB module enables the aggregation of local and global contextual features with specific emphasis on enhancing representations along horizontal and vertical orientations. This targeted enhancement substantially boosts the network’s ability to model directionality and maintain spatial continuity, thereby improving the detection and structural understanding of cracks under complex and visually challenging conditions.

In the upsampling stage, we replace all traditional upsampling operations, such as nearest neighbor or bilinear interpolation, with the Dynamic Sampling Upsampling Block (DSUB) [35]. Conventional upsampling methods typically employ fixed, content-independent sampling strategies, which often result in blurred crack boundaries and the loss of fine structural details—issues that are particularly pronounced for small or low-contrast cracks under adverse weather. The DSUB module addresses these challenges by predicting adaptive offsets and scales for each spatial position, thus enabling content-aware dynamic sampling that accurately reconstructs feature details and preserves essential edge information. This targeted approach significantly enhances the recovery of spatial details during upsampling, yielding sharper crack boundaries and more precise localization, even in visually degraded or complex real-world scenarios.

Our improved network first extracts features using the HWDB-augmented backbone, then fuses multi-scale and directional features with SPBB modules, and finally restores spatial details with the DSUB module before outputting high-quality crack detection and classification results. Each structural innovation we design closely corresponds to a key challenge in crack detection under adverse weather conditions, ensuring both theoretical soundness and practical robustness.

### 3.2. Strip Pooling Bottleneck Block

In adverse weather conditions such as rain and snow, the visibility of pavement cracks is significantly reduced. Cracks often appear as elongated, fragmented, or low-contrast structures, sometimes partially occluded, which greatly increases the difficulty of automatic detection. The main challenges lie in the fact that conventional convolutional architectures have limited capacity for global context modeling and long-range dependency, making it difficult to accurately detect or recover cracks that are blurred or occluded. Moreover, cracks usually exhibit strong directional patterns (e.g., horizontal or vertical extension), while ordinary networks struggle to effectively capture such directional information. Therefore, designing an efficient feature extraction module that can aggregate multi-scale context, directional information, and global semantics is critical to improve crack detection performance under adverse weather.

To address these issues, we introduce the Strip Pooling Bottleneck Block (SPBB), as illustrated in Figure 2b. This block utilizes multi-branch aggregation and double residual connections to efficiently integrate global, local, and direction-aware features, significantly enhancing the robustness and representation power of the network for complex pavement crack scenarios. The detailed process is as follows:

Given the input feature *X*, a 3×3 convolution is first applied to extract spatial structure information, resulting in feature *F*. Then, a 1×1 convolution is performed for channel adjustment and feature mixing, and the result is used as input to the upper and lower branches, which are denoted as $F_{1}$ and $F_{2}$:

(1) $$F={\mathrm{Conv}}_{3\times 3}\left(X\right)$$

(2) $$F_{1}={\mathrm{Conv}}_{1\times 1}\left(F\right)$$

(3) $$F_{2}={\mathrm{Conv}}_{1\times 1}\left(F\right)$$

where *X* is the input feature map, *F* is the output after the 3×3 convolution, ${\mathrm{Conv}}_{3\times 3}\left(\cdot \right)$ and ${\mathrm{Conv}}_{1\times 1}\left(\cdot \right)$ denote 3×3 and 1×1 convolution operations, and $F_{1}$, $F_{2}$ are the inputs to the upper and lower branches, respectively.

In the upper branch, $F_{1}$ is passed through three sub-paths. The first and third sub-branches perform global average pooling (GAP) followed by 3×3 convolution, while the second sub-branch applies a 3×3 convolution directly. The outputs are then concatenated along the channel dimension to produce $F_{up}$:

(4) $$F_{g1}={\mathrm{Conv}}_{3\times 3}\left(\mathrm{GAP}\left(F_{1}\right)\right)$$

(5) $$F_{l}={\mathrm{Conv}}_{3\times 3}\left(F_{1}\right)$$

(6) $$F_{g3}={\mathrm{Conv}}_{3\times 3}\left(\mathrm{GAP}\left(F_{1}\right)\right)$$

(7) $$F_{up}=\mathrm{Concat}\left(F_{g1},F_{l},F_{g3}\right)$$

where GAP(·) denotes global average pooling, $F_{g1}$ and $F_{g3}$ are global context features, $F_{l}$ is the local detail feature, Concat(·) indicates channel concatenation, and $F_{up}$ is the output of the upper branch.

In the lower branch, $F_{2}$ is processed by horizontal and vertical strip pooling, i.e., average pooling along the width and height, respectively, which are each followed by 3×3 convolution. The resulting features are then concatenated to obtain $F_{low}$:

(8) $$F_{h}={\mathrm{Conv}}_{3\times 3}\left({\mathrm{AvgPool}}_{1\times W}\left(F_{2}\right)\right)$$

(9) $$F_{v}={\mathrm{Conv}}_{3\times 3}\left({\mathrm{AvgPool}}_{H\times 1}\left(F_{2}\right)\right)$$

(10) $$F_{low}=\mathrm{Concat}\left(F_{h},F_{v}\right)$$

where ${\mathrm{AvgPool}}_{1\times W}\left(\cdot \right)$ and ${\mathrm{AvgPool}}_{H\times 1}\left(\cdot \right)$ denote average pooling along the height and width, respectively; $F_{h}$ and $F_{v}$ are the features aggregated in the horizontal and vertical directions, and $F_{low}$ is the output of the lower branch.

The outputs of the upper and lower branches are concatenated along the channel dimension to form $F_{cat}$, which is then added to the output *F* of the first 3×3 convolution to obtain the first residual output $F_{res1}$:

(11) $$F_{cat}=\mathrm{Concat}\left(F_{up},F_{low}\right)$$

(12) $$F_{res1}=F_{cat}+F$$

where $F_{cat}$ is the concatenated multi-branch feature, *F* is the 3×3 convolution output, and $F_{res1}$ is the first residual output.

Finally, the first residual output $F_{res1}$ is added to the original input *X* to obtain the final output *Y* of the SPB module:

(13) $$Y=F_{res1}+X$$

where $F_{res1}$ is the output after the first residual connection, *X* is the input feature, and *Y* is the final output of the SPB module.

In summary, the SPBB module effectively integrates global context, local spatial information, and direction-aware features, significantly improving the network’s ability to detect elongated, fragmented, low-contrast, and strongly directional cracks. The dual residual connections ensure sufficient information flow and feature diversity throughout the module, providing a robust and expressive high-level semantic feature for subsequent detection heads. Overall, the proposed SPB module lays a solid foundation for crack detection in extreme environments and greatly enhances the model’s generalization capability in complex scenarios.

### 3.3. Haar Wavelet Downsampling Block (HWDB)

Conventional downsampling techniques, such as max or average pooling, inevitably result in the loss of critical spatial details and high-frequency information—an issue particularly detrimental for detecting small, faint, or low-contrast pavement cracks in adverse weather conditions such as rain and snow. Under these circumstances, cracks often exhibit blurred contours, weak intensity, and are prone to occlusion or distortion by environmental noise, as shown in Figure 2a. Preserving both global structure and fine-grained edge cues during downsampling is thus essential for robust and reliable crack detection in real-world outdoor scenes.

To address these challenges, we introduce an information-preserving the Haar Wavelet Downsampling Block (HWDB), as illustrated in Figure 2c. Rather than reducing spatial resolution through direct aggregation, the HWDB module leverages an orthogonal frequency decomposition to retain vital spatial information. Specifically, for each 2×2 spatial region in the input feature map *X*, the HWDB module computes one low-frequency and three high-frequency components according to

(14) $$\begin{array}{cc} Y_{LL} & =\frac{1}{2}\left(x_{1}+x_{2}+x_{3}+x_{4}\right),\\ Y_{LH} & =\frac{1}{2}\left(x_{1}-x_{2}+x_{3}-x_{4}\right),\\ Y_{HL} & =\frac{1}{2}\left(x_{1}+x_{2}-x_{3}-x_{4}\right),\\ Y_{HH} & =\frac{1}{2}\left(x_{1}-x_{2}-x_{3}+x_{4}\right), \end{array}$$

where $x_{1},x_{2},x_{3},x_{4}$ are the values of each 2×2 patch in *X*, $Y_{LL}$ denotes the low-frequency (approximation) coefficient, and $Y_{LH},Y_{HL},Y_{HH}$ represent horizontal, vertical, and diagonal high-frequency components, respectively.

These four sub-bands, each retaining the spatial dimensions H×W and *C* channels, are concatenated along the channel axis to form a frequency-aware feature representation:

(15) $$F_{cat}=\mathrm{Concat}\left(Y_{LL},\;Y_{LH},\;Y_{HL},\;Y_{HH}\right),$$

with $F_{cat}\in R^{H\times W\times 4C}$. To further promote adaptive feature fusion and dimensionality reduction, the HWDB module employs a 1×1 convolution followed by batch normalization and a ReLU activation:

(16) $$F_{out}=\mathrm{ReLU}\left(\mathrm{BN}\left({\mathrm{Conv}}_{1\times 1}\left(F_{cat}\right)\right)\right),$$

where ${\mathrm{Conv}}_{1\times 1}\left(\cdot \right)$ projects 4C channels back to *C*, BN(·) denotes batch normalization, and ReLU(·) is the activation function. The resulting $F_{out}$ serves as the downsampled feature, preserving both global context and high-frequency edge cues vital for subsequent crack detection stages.

This orthogonal transform enables the HWDB module to capture and transmit spatial structure, boundary information, and textural details that would otherwise be lost in traditional downsampling. By systematically integrating the HWDB module into all downsampling layers of our detection backbone, the network gains the capacity to robustly localize and delineate cracks—even under heavy rain, snow, and low-visibility conditions—thereby providing a reliable and information-rich feature foundation tailored for adverse-weather pavement crack detection.

### 3.4. Dynamic Sampling Upsampling Block (DSUB)

Under complex conditions such as rain and snow, pavement cracks often exhibit elongated, fragmented, and low-contrast characteristics, making the spatial structural recovery of feature upsampling crucial for accurate detection. Traditional upsampling methods, such as bilinear interpolation, cannot adaptively adjust sampling locations based on content, leading to blurred edges and the loss of details. As shown in Figure 2d, to address this, we introduce the Dynamic Sampling Upsampling Block into our method, which implements content-adaptive dynamic upsampling and effectively enhances the recovery of structural and fine details.

Specifically, given an input feature map $X_{Input}\in R^{B\times C\times H\times W}$, we first use convolutional branches to predict the offset *O* and scale *S* for each spatial location as

(17) $$O=Conv_{offset}\left(X_{Input}\right),\;S=Conv_{scale}\left(X_{Input}\right)$$

where *O* is the output of the offset branch with shape [B,2,H,W], and *S* is the output of the scale branch with shape [B,1,H,W] or [B,2,H,W].

Then, the offset and scale are multiplied element-wise to obtain a content-adaptive scaled offset:

(18) $$O^{\prime }=O\times S$$

where $O^{\prime }$ represents the adaptively scaled offset with the same shape as *O*.

Next, $O^{\prime }$ is added to the initial reference grid init_pos of the target space to produce the content-adaptive sampling points:

(19) $$P=O^{\prime }+init\text{\_}pos$$

where init_pos denotes the standard sampling positions for each pixel in the target space (typically generated by meshgrid and normalized to [−1,1]).

The sampling points *P* are then reshaped to match the target high-resolution space. After that, the reshaped *P* is added to the standard coordinate grid coords of the target space, yielding the final dynamic sampling coordinates:

(20) $$G=P_{reshape}+coords$$

where coords indicates the standard coordinate grid for the upsampled output space with shape [B,2,sH,sW].

Afterwards, the features are passed through a pixel shuffle operation to reorganize spatial and channel information:

(21) $$F_{shuffled}=pixel\text{\_}shuffle\left(X_{Input}\right)$$

Finally, the grid_sample function is employed, using *G* as the sampling coordinates to perform dynamic content-aware sampling on the input features, thereby producing the final high-resolution output:

(22) $$X_{Output}=grid\text{\_}sample\left(F_{shuffled},G\right)$$

where pixel_shuffle(·) denotes the pixel shuffle operation, $F_{shuffled}$ is the feature map after shuffling, and $X_{Output}$ is the final output with shape [B,C,sH,sW].

By following this content-adaptive dynamic sampling process, the introduced DySample module allows each upsampled pixel to extract features from the optimal spatial location, substantially enhancing the recovery of spatial details and edge information. This provides a robust feature foundation for high-resolution crack detection in complex environments.

## 4. Experiment

### 4.1. Datasets and Evaluation Metrics

**Figure 3:**
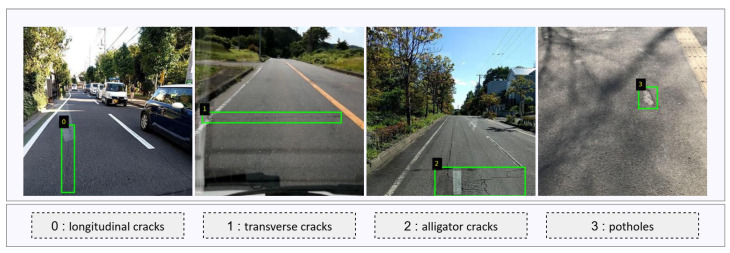
Representative image samples of the four pavement defect categories in the dataset: (**0**) longitudinal cracks; (**1**) transverse cracks; (**2**) alligator cracks; (**3**) potholes. The green bounding boxes denote the annotated defect regions.

To comprehensively evaluate the robustness and generalization ability of our method in complex real-world environments, we construct three datasets based on the RDD2022 [29] Japan subset. First, the original RDD2022 Japan dataset, which contains a variety of pavement types and multiple crack categories (such as longitudinal cracks, transverse cracks, alligator cracks, and potholes), collected under normal weather conditions, is used as the clear-weather baseline. In addition to the rain/snow-augmented Japan dataset, we introduce the Norway subset of RDD2022 as an independent test set to assess the generalization performance of our model. The Norway dataset was collected by the Norwegian Public Road Administration using high-resolution vehicle-mounted cameras and covers both expressways and county roads. Notably, the Norway images capture a wide range of post-rain, snow-covered, and overcast conditions, closely resembling the challenging real-world scenarios our method is designed to address. As highlighted in the original RDD2022 paper, the inclusion of the Norway subset enhances the dataset’s diversity and provides an excellent testbed for evaluating model robustness and transferability across geographical regions and adverse environmental conditions. Table 1 summarizes the key statistics of the two datasets used in this study, including the number of training and validation images and the number of annotated bounding boxes for each defect category. Figure 3 shows representative image samples from each of the four major defect classes, providing a clear visualization of the differences in appearance and annotation style.

To simulate realistic rainy conditions, we design a procedural rain synthesis pipeline that overlays multiple layers of randomly distributed lines with varying lengths and angles onto the original images, simulating rain streaks of different intensity and direction. Each layer of rain streaks is further enhanced by applying motion blur along the main rain direction, resulting in a more natural dynamic effect. The rain layers are then fused with the original image using adjustable transparency to control the severity of the rain effect. By adjusting the relevant parameters, we are able to synthesize a wide range of rainfall scenarios, from light drizzle to heavy downpour, providing challenging samples for crack detection under various precipitation intensities. Finally, for snowy conditions, we employ a two-stage synthesis approach. First, we generate a transparent snowflake mask for each image, consisting of thousands of randomly distributed snow particles with variable shapes, sizes, transparency, and trailing effects, capturing the spatial layering and depth of real snowfall. The snow mask is then blended with the clear-weather image at a specified transparency level, resulting in realistic snow occlusion where cracks and pavement may be partially covered. All datasets are split into training and validation sets at a ratio of 10:3, ensuring a balanced distribution of crack categories and weather conditions and providing a solid foundation for the evaluation of our detection framework.

For evaluation, we adopt several standard metrics to assess the effectiveness of our method in pavement crack detection, including Precision, mAP@0.5, mAP@0.5:0.95, and category-wise average precision for each type of crack. Precision measures the proportion of correctly predicted crack instances among all positive detections, reflecting the ability of model to reduce false positives. In addition, we further used precision–recall (PR) curves to compare the performance of the baseline model and the proposed CrackNet-Weather at different IoU thresholds to better characterize the stability and recall performance in low-contrast scenarios and the fine crack detection of the model. mAP@0.5 is a widely used benchmark in object detection, while mAP@0.5:0.95 provides a comprehensive evaluation by averaging performance across multiple IoU thresholds. In addition to the overall results, we also report the average precision for each individual crack category, including longitudinal cracks, transverse cracks, alligator cracks, and potholes, to evaluate the detection accuracy for different defect types. We further report the number of model parameters to indicate model size and resource requirements, and we use GFLOPs to reflect computational complexity and efficiency. All experimental results are evaluated on the validation set to ensure a fair and objective comparison.

### 4.2. Implementation Details

All experiments were conducted using the Ultralytics YOLOv12 framework on a single NVIDIA RTX 3090 GPU.(NVIDIA Corporation, Santa Clara, CA, USA) During training, the model was trained for 220 epochs with a batch size of 32, and all input images were resized to 640 × 640 pixels. The optimizer used was stochastic gradient descent (SGD) with the default learning rate as provided by the official implementation. To improve the generalization capability of the model, a range of data augmentation strategies were employed during training, including scale augmentation (with the scale factor set to 0.5), mosaic augmentation (set to 1.0), and copy-paste augmentation (set to 0.1); mixup augmentation was not used. Mixed precision training (AMP) was disabled to ensure stable convergence. The dataset was organized in COCO format, and the ratio of training set to validation set was 10:3. Unless otherwise specified, all other hyperparameters and training procedures followed the official Ultralytics YOLOv12 implementation.

### 4.3. Ablation Studies

To systematically evaluate the effectiveness of each proposed module, we conducted ablation experiments based on the YOLOv12-s. We analyzed the impact of the HWDB [33], SPBB [34], and DSUB [35] modules, both individually and in combination, on detection accuracy and model complexity. The experiments were performed under two adverse weather conditions (rain and snow) and covered four types of pavement cracks. All of the detection results reported in Table 2 are mAP@0.5:0.95 in percentage form. The corresponding computational complexity (FLOPs) and number of parameters for each configuration are listed in Table 3.

**Effect of the Strip Pooling Bottleneck Block (SPBB)**. After incorporating the Strip Pooling Bottleneck Block (SPBB), the overall detection accuracy (mAP) increases significantly: from 16.0 to 17.0 under rain and from 15.1 to 16.3 under snow. Notably, the performance for transverse and alligator cracks under rain is markedly enhanced with the mAP for transverse cracks increasing from 7.5 to 8.8 and for alligator cracks from 27.0 to 28.8. While accuracy improves, the FLOPs rise moderately from 21.5 G to 29.5 G, and the parameter count slightly decreases from 9.25 M to 9.10 M. This indicates that the SPBB module enhances the ability of model to capture directional and long-range contextual features with only a slight reduction in computational efficiency. Considering that real-world road monitoring often involves many elongated, directional, and occluded cracks, the SPBB module greatly improves the robustness of the model in complex and challenging environments.

**Effect of the Haar Wavelet Downsampling Block (HWDB)**. The Haar Wavelet Downsampling Block (HWDB) also yields outstanding results with the mAP rising to 17.3 under rain and 16.2 under snow. The most significant gain appears in the detection of potholes, where the mAP increases from 16.0 to 18.1 under rain. Importantly, the HWDB module drastically reduces the number of model parameters (from 9.25 M to 8.19 M) and lowers FLOPs to 18.9 G, making the model more lightweight. This demonstrates that the HWDB module can effectively retain important structural information during downsampling while considerably reducing model complexity, enhancing the detection of low-contrast and weak-texture cracks. The HWDB module is thus well suited for resource-constrained or large-scale road monitoring applications.

**Effect of the Dynamic Sampling Upsampling Block (DSUB)**. The Dynamic Sampling Upsampling Block (DSUB) primarily improves the recovery of fine-grained structural information, especially for transverse cracks, where the mAP under rain increases substantially from 7.5 to 10.4. The overall mAP reaches 16.9/15.9 (rain/snow). The introduction of the DSUB has a negligible effect on FLOPs (21.3 G) and does not increase the parameter count (9.25 M). This shows that the DSUB, by means of content-adaptive spatial feature reconstruction, greatly enhances the perception of crack boundaries and small objects in complex backgrounds for the model while maintaining practical inference speed. This capability is essential for the timely detection of early-stage pavement crack.

In conclusion, the systematic ablation study demonstrates that the proposed three modules not only lead to significant improvements in detection accuracy across different crack categories and adverse weather conditions but also maintain a well-balanced computational complexity. Specifically, the SPBB, HWDB, and DSUB modules each provide targeted structural enhancements for challenging crack features such as occlusions, low contrast, and fine or fragmented patterns that frequently occur in real-world road environments. Regardless of the type—longitudinal, transverse, alligator, or potholes—these innovative modules show strong adaptability and robustness, meeting the dual demands for complex scene recognition and efficient inference in practical road monitoring. Importantly, the overall model maintains a reasonable parameter size and FLOPs while achieving improved accuracy, which is beneficial for deployment on edge devices or in resource-constrained engineering scenarios. Taken together, the experimental results confirm that the improved architecture proposed in this study offers a more reliable and efficient solution for pavement crack detection under adverse weather and complex scene conditions.

### 4.4. Comparisons

To further validate the effectiveness and generalization capability of our proposed method in the field of pavement crack detection, we conducted comparisons with several representative mainstream object detection models, including YOLOv8-s, YOLOv10-s, YOLOv11-s, YOLOv12-s, and YOLOv13-s. All models were trained and evaluated on the same rainy and snowy pavement crack detection dataset. The experimental results are presented in Table 4, where mAP@0.5:0.95, FLOPs, and the number of parameters comprehensively reflect the detection ability and model complexity.

As shown in Table 4, our method achieves the best overall detection performance under both rainy and snowy conditions with an average mAP of 17.8 (rain) and 16.8 (snow). Compared to common baselines such as YOLOv8-s and YOLOv12-s, our method improves the mAP by 0.9/0.8 and 1.8/1.7 points (rain/snow), respectively. For the challenging alligator cracks and potholes categories, which are difficult due to their complex shapes and low contrast, our method achieves mAP values of 29.7/29.3 and 19.1/17.0, respectively, demonstrating a more pronounced advantage over other models.

In terms of computational complexity, although the FLOPs (26.9 G) of our method are slightly higher than those of YOLOv8-s and YOLOv12-s, the parameter count is only 8.05 M, which is the lowest among all of the compared models. Considering the significant improvement in detection accuracy achieved by our method, this moderate increase in FLOPs is acceptable. Overall, our method can effectively improve the detection accuracy of pavement cracks under adverse weather and complex pavement conditions while maintaining reasonable model complexity. The model is suitable for road inspection scenarios with high accuracy requirements and also takes into account inference speed and resource consumption, which offers clear advantages for addressing challenges such as environmental interference and hard-to-detect targets in real-world road monitoring. These results further demonstrate the practicality and application potential of our method in the field of intelligent pavement crack detection.

To further verify the generalization capability of the proposed method, we conducted additional experiments on the Norway pavement defect dataset. The comparison results with mainstream detection models are summarized in Table 5. Our method (CrackNet) achieves an AP _50:95_ of 9.22% and an AP_50_ of 24.24%, which outperforms YOLOv8-s, YOLOv11-s, YOLOv12-s, and YOLOv13-s, and these results are comparable to or better than the two-stage models Faster R-CNN and Cascade. In particular, CrackNet surpasses YOLOv12-s by 1.83 percentage points in AP_50:95_ and 5.42 percentage points in AP_50_, demonstrating superior generalization performance on an unseen dataset.

Meanwhile, CrackNet maintains a lower computational complexity with only 26.91 GFLOPs and 8.05 M parameters, which is significantly less than Faster R-CNN (151.31 GFLOPs, 28.30 M) and Cascade (231.00 GFLOPs, 69.16 M). These results indicate that our method achieves a good trade-off between detection accuracy and computational cost, and it possesses strong generalization ability across different datasets and scenarios.

To provide a more comprehensive evaluation, we further examined the performance of CrackNet-Weather through precision–recall (PR) curves under different IoU thresholds. Specifically, we compared the baseline model with our proposed model across two thresholds and two datasets (rainy and snowy conditions) in order to highlight their differences in precision–recall trade-offs.

**Figure 4:**
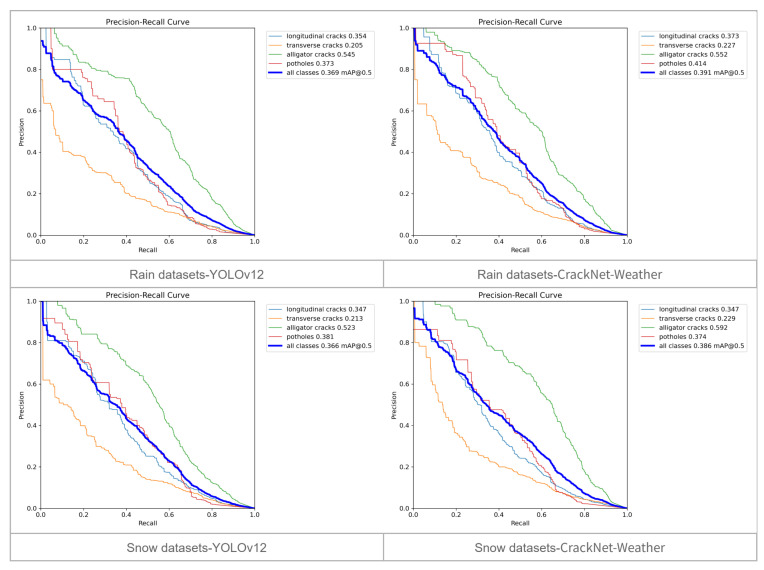
Precision–recall (PR) plot with an IoU of 0.5. The (**top**) half shows the PR plot for the rain dataset, while the (**bottom**) half shows the PR plot for the snow dataset. The (**left**) side shows the baseline model, and the (**right**) side shows CrackNet-Weather.

When the IoU threshold is 0.5, as shown in Figure 4, CrackNet-Weather shows clear improvements in recall across both rainy and snowy datasets. The PR curves consistently shift toward the top-right region compared with YOLOv12, which indicates that the model can capture a larger proportion of positive instances while sustaining comparable precision. Importantly, the gains are most evident for longitudinal and transverse cracks, which are more likely to appear faint and fragmented in rainy or snowy conditions. This suggests that the proposed model is more capable of suppressing missed detections for low-contrast crack types, which is a factor that directly enhances safety relevance.

**Figure 5:**
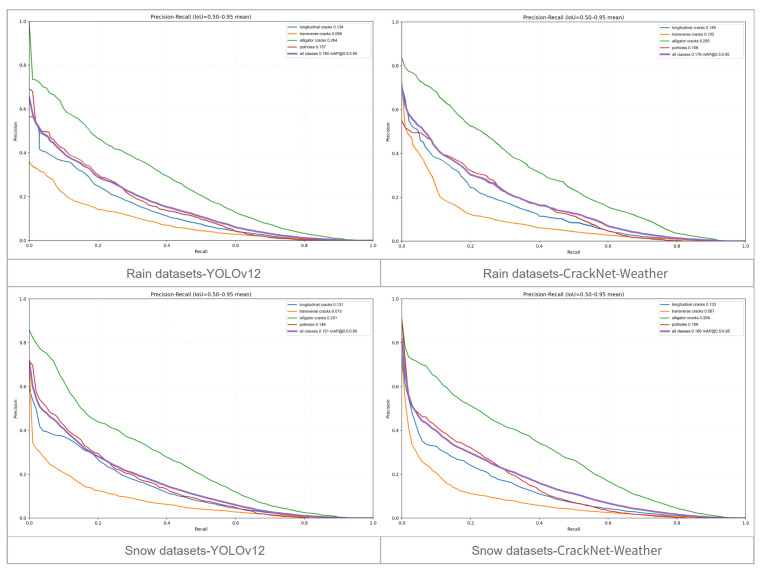
Precision-recall (PR) plots for IoU ranges of 0.5-0.95. The (**top**) half shows the PR plot for the rain dataset, while the (**bottom**) half shows the PR plot for the snow dataset. The (**left**) side shows the baseline model, and the (**right**) side shows CrackNet-Weather.

When the IoU threshold is 0.5–0.95, as shown in Figure 5, the evaluation emphasizes not only whether the crack is detected but also whether the crack is accurately localized. Under this stricter criterion, CrackNet-Weather still maintains a larger area under the PR curve compared to YOLOv12 in most categories. This advantage is particularly evident in cracks and potholes, which have irregular shapes and blurred edges in inclement weather, making accurate localization difficult. CrackNet-Weather’s superior stability under these conditions highlights its strong feature representation and localization capabilities, ensuring reliable detection even under challenging alignment requirements.

The dual-level PR analysis confirms that CrackNet-Weather provides benefits beyond higher mAP scores. It improves the recall of fine and low-contrast cracks at lower IoU thresholds, reducing the likelihood of critical missed detections. At the same time, it sustains robust localization accuracy at stricter thresholds, reflecting a stronger generalization ability under diverse weather scenarios.

### 4.5. Model Robustness Verification

To rigorously evaluate the robustness and generalization ability of CrackNet-Weather, we constructed two balanced test subsets: a Normal Weather Dataset (NWD) and an Extreme Weather Dataset (EWD). Specifically, we randomly selected 2000 images from the original Japan dataset to represent the normal weather scenario, and then we selected another 2000 images from our synthesized snow datasets to represent extreme weather conditions. In both subsets, we ensured that the distribution of defect types was balanced, so that each major defect category is equally represented. This design guarantees an objective and fair comparison of model performance across different environmental conditions.

**Figure 6:**
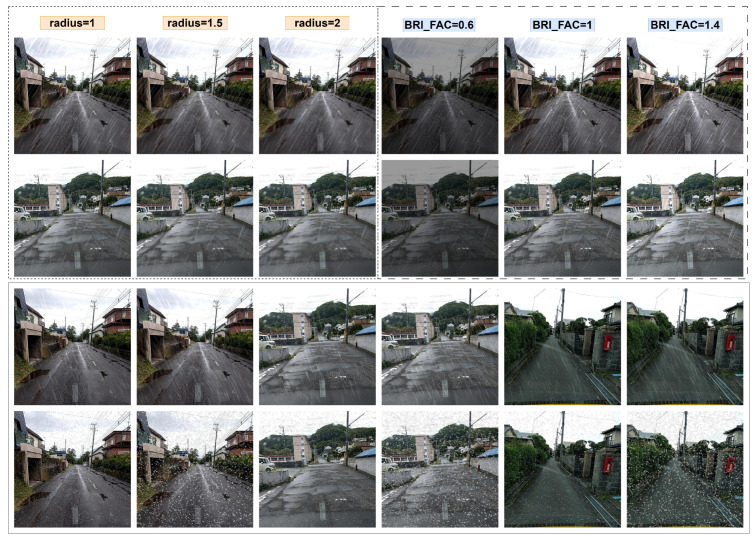
Examples of image degradation used for robustness verification. The first two rows illustrate Gaussian blur (radius = 1, 1.5, 2) and brightness variation (factors 0.6, 1.0, 1.4). The last two rows show synthetic rain and snow generated by our augmentation algorithms, where each pair of images represents light (**left**) and heavy (**right**) conditions.

As illustrated in Figure 6, in addition to the comparison under normal and extreme weather subsets, we further verified the robustness of the models under parameterized visual degradations. Specifically, three levels of localized Gaussian blur (radius = 1, 1.5, 2) were applied to crack regions to simulate the partial blurring of pavement defects under rainy or snowy conditions, while brightness factors of 0.6, 1.0, and 1.4 were used to emulate dark, normal, and bright illumination scenarios. It is worth noting that both the blur and brightness variations were applied on top of the normal rain subset to simulate additional imaging degradations. Furthermore, light and heavy rain/snow conditions were synthesized using our augmentation algorithms (Algorithms 1 and 2) with explicit parameter settings: light rain (*Nlayers* = 1, *Nlines* = 400, *kmb* = 5, α = 0.15), heavy rain (*Nlayers* = 3, *Nlines* = 1200, *kmb* = 9, α = 0.25), light snow (*Nsnow* = 3k, *size* = [1, 3], $\alpha_{mask}$ = 0.15, streak = 20%), and heavy snow (*Nsnow* = 10k, *size* = [3, 6], $\alpha_{mask}$ = 0.25, streak = 50%). These controlled perturbations enable a systematic and reproducible evaluation of model robustness across diverse degradation scenarios, ensuring that the observed performance differences are not limited to a single weather condition but generalize across a spectrum of visual interferences.

Table 6 presents the performance of both models across different weather conditions. Under normal weather conditions (NWD), the baseline model YOLOv12 achieved an AP_50–95_ value of 18.0% and an AP_50_ value of 40.4%. In comparison, the proposed CrackNet achieved AP_50–95_ and AP_50_ values of 19.2% and 43.2%, respectively, indicating improved detection capability under ideal conditions. However, under extreme weather conditions (EWD), CrackNet demonstrated significant robustness advantages. Specifically, CrackNet achieved an AP_50–95_ value of 16.8%, representing a 1.7% improvement over the baseline model. For the AP_50_ metric, CrackNet reached 38.3%, outperforming the baseline model’s 34.3%. These results indicate that the proposed method can better handle visual interference and feature degradation caused by adverse weather such as rain and snow, and they demonstrate its potential for deployment in challenging real-world scenarios that require reliable pavement defect detection and automated road inspection.

Table 7 summarizes CrackNet’s performance under a spectrum of weather- and imaging-induced degradations. A consistent trend emerges across conditions: as the severity of the degradation increases, detection performance decreases. In particular, heavier rain/snow introduces denser streaks and occlusions that suppress edge contrast along crack boundaries, leading to larger drops in both ${\mathrm{AP}}_{50–95}$ and ${\mathrm{AP}}_{50}$. For localized Gaussian blur applied on the Normal (Rain) subset, the metrics degrade monotonically with the blur radius, reflecting the model’s sensitivity to a loss of high-frequency cues that are critical for accurate crack localization. The brightness variation further shows that under-exposure (darker scenes) is more detrimental than over-exposure, which is consistent with the reduction of signal-to-noise ratio and diminished crack–background separability under low illumination.

Notably, ${\mathrm{AP}}_{50–95}$ exhibits larger relative drops than ${\mathrm{AP}}_{50}$ across all adverse settings, indicating that degradations affect precise localization at higher IoU thresholds more than coarse detection at 0.5 IoU. Despite these challenges, CrackNet maintains stable performance trends across diverse perturbations and preserves competitive ${\mathrm{AP}}_{50}$ values, evidencing the robustness of its feature representations and decision head. The consistent ranking of difficulty (normal > light > heavy; small *r* > large *r*; normal/bright > dark) and the controlled, parameterized construction of the subsets further suggest that the model’s behavior generalizes systematically rather than overfitting to a particular weather type. Taken together, the results indicate that CrackNet retains reliable detection capacity across parameterized adverse-weather and imaging degradations, supporting its use in non-ideal, real-world pavement inspection.

To quantitatively evaluate robustness under different weather severities and imaging degradations, we conducted graded tests on *light/normal/heavy* rain and snow, and we further tested under *partial Gaussian blur* and *brightness variation* (under-/over-exposure). Table 6 reports the AP_50:95_ and AP_50_ values on the RDD2022-Japan subset.
**Algorithm 1** Pseudocode for Procedural Snowfall Augmentation**Input:** Original image $I_{orig}$Image directory *D* (for batch processing)Number of snowflakes $N_{snow}$Mask transparency $\alpha_{mask}$Snow particle size range $\left[s_{min},s_{max}\right]$Snow mask output path $M_{out}$**Output:** Snow-augmented image $I_{snow}$**Step 1: Generate Snow Mask** 1.Create an empty RGBA mask *M* with the same size as $I_{orig}$.2.For i=1 to $N_{snow}$: Randomly sample position $\left(x_{i},y_{i}\right)$ in the image.Randomly select particle type: grain or streak.If grain: –Sample radius $r_{i}$ from $\left[s_{min},s_{max}\right]$.–Sample alpha $\alpha_{i}$ from $\left[\alpha_{min},\alpha_{max}\right]$.–Draw an irregular filled polygon at $\left(x_{i},y_{i}\right)$ with $r_{i}$ and $\alpha_{i}$ on *M*.If streak: –Sample length $l_{i}$ from $\left[2.5r_{i},7.0r_{i}\right]$, width $w_{i}$ from [0.8,1.4].–Sample angle $\theta_{i}$ from $\left[-\frac{\pi }{4},\frac{\pi }{4}\right]$.–Sample alpha $\alpha_{i}$ from $\left[\alpha_{min},\alpha_{max}\right]$.–Draw a line at $\left(x_{i},y_{i}\right)$ of length $l_{i}$, width $w_{i}$, angle $\theta_{i}$, $\alpha_{i}$ on *M*.3.Apply Gaussian blur (e.g., σ=0.6) to *M* for realism.4.Save *M* as a snow mask to $M_{out}$.**Step 2: Blend Snow Mask with Original Image** 1.For each image $I_{orig}$ in directory *D*: Resize *M* to match $I_{orig}$ if needed.For each pixel (x,y): –Compute blended pixel:$I_{snow}\left(x,y\right)=\left(1-\alpha_{mask}\right)\cdot I_{orig}\left(x,y\right)+\alpha_{mask}\cdot M_{RGB}\left(x,y\right)$Save $I_{snow}$ to $M_{out}$.2.Output the snow-augmented images.

**Algorithm 2** Pseudocode for Procedural Rainfall Augmentation
**Input:**

Original image $I_{orig}$Image directory *D* (for batch processing)Number of rain layers $N_{layers}$Number of rain lines per layer $N_{lines}$Rain line length range $\left[l_{min},l_{max}\right]$Rain line angle mean $\mu_{\theta }$ and std $\sigma_{\theta }$Rain line thickness range $\left[w_{min},w_{max}\right]$Rain line brightness range $\left[b_{min},b_{max}\right]$Motion blur kernel size $k_{mb}$Transparency (per-layer alpha) range $\left[\alpha_{min},\alpha_{max}\right]$Output path $O_{out}$
**Output:**

Rain-augmented image $I_{rain}$
**Step 1: Generate and Overlay Rain Layers**

1.Initialize $I_{rain}=I_{orig}$2.**For** j=1 to $N_{layers}$: Create empty mask $M_{j}$ with same size as $I_{orig}$**For** i=1 to $N_{lines}$: –Randomly sample start point $\left(x_{start},y_{start}\right)$–Randomly sample length $l_{i}\in \left[l_{min},l_{max}\right]$–Randomly sample angle $\theta_{i}∼N\left(\mu_{\theta },\sigma_{\theta }\right)$–Compute end point $\left(x_{end},y_{end}\right)$ by $l_{i}$ and $\theta_{i}$–Randomly sample thickness $w_{i}\in \left[w_{min},w_{max}\right]$–Randomly sample brightness $b_{i}\in \left[b_{min},b_{max}\right]$–Draw line from $\left(x_{start},y_{start}\right)$ to $\left(x_{end},y_{end}\right)$ with width $w_{i}$, brightness $b_{i}$ on $M_{j}$Apply motion blur with kernel size $k_{mb}$ along $\theta_{j}$ to $M_{j}$Randomly sample transparency $\alpha_{j}\in \left[\alpha_{min},\alpha_{max}\right]$Blend $M_{j}$ onto $I_{rain}$:$I_{rain}=\left(1-\alpha_{j}\right)\cdot I_{rain}+\alpha_{j}\cdot M_{j}$
**Step 2: Batch Processing and Output**

1.Repeat the above for each $I_{orig}$ in directory *D*2.Save $I_{rain}$ to $O_{out}$3.Output the rain-augmented images


To further characterize stability, we summarize each factor (rain, snow, blur, brightness) with two severity-aware metrics:

(23) $$\mathrm{Normalized}\;\mathrm{Drop}\;=\;\frac{AP_{\mathrm{Light}}-AP_{\mathrm{Heavy}}}{AP_{\mathrm{Light}}}\times 100\%,$$

(24) $$\mathrm{Severity}\;\mathrm{Slope}\;=\;\frac{AP_{\mathrm{Heavy}}-AP_{\mathrm{Light}}}{2}\;\left(\mathrm{pp}/\mathrm{level}\right).$$

where $AP_{\mathrm{Light}}$, $AP_{\mathrm{Normal}}$, and $AP_{\mathrm{Heavy}}$ denote the overall AP_50:95_ measured on the Light, Normal, and Heavy subsets of the same factor; “pp” denotes percentage points. For brightness, severities are ordered as *over-exposed (Light)* → *normal (Normal)* → *under-exposed (Heavy)*.

Table 8 condenses the severity-wise behavior of our detector using AP_50:95_. Across all factors, the *Heavy*-level AP remains a large fraction of the *Light*-level AP—85.5% for rain, 82.8% for snow, 90.7% for blur, and 87.7% for brightness—placing the worst-case loss within a narrow band (Normalized Drop 9.26–17.23%). The *per-level* degradation is also modest: the Severity Slope lies between −0.78 and −1.49 pp/level with *snow* being the hardest (17.23%, −1.49) and *blur* the mildest (9.26%, −0.78); *rain* sits in between (14.51%, −1.335), while *brightness* is moderate (12.29%, which is −1.045), consistent with the noted asymmetry that under-exposure harms more than over-exposure. Taken together—*bounded worst-case loss* and *small, nearly constant per-level loss*—these numbers show that accuracy degrades *gradually and predictably* as conditions worsen; i.e., the model is stable under graded adverse weather and imaging degradations.

## 5. Visualization

**Figure 7:**
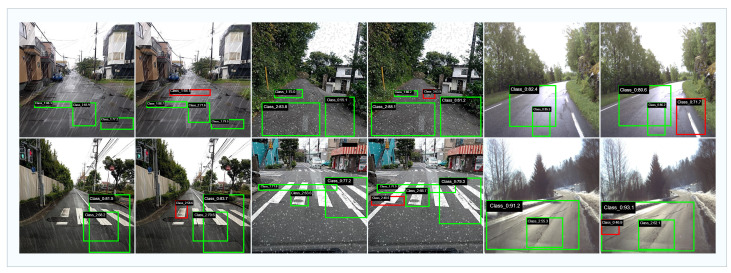
Qualitative visualization of detection results for YOLOv12 (**left**) and CrackNet- Weather (**right**) under adverse weather conditions. Green bounding boxes denote correctly detected pavement defects by each model. Red bounding boxes indicate ground-truth defects that were missed by YOLOv12 but successfully detected by CrackNet-Weather. These results demonstrate the superior ability of CrackNet-Weather to identify subtle and challenging defects, especially under severe visual interference.

To further demonstrate the advantages of the proposed method, Figure 7 shows a qualitative comparison of detection results under challenging weather conditions. In each example, the left image displays the predictions of the baseline YOLOv12 model, while the right image shows those of CrackNet-Weather on the same scene. Green bounding boxes denote pavement defects that are correctly detected by each model. Red bounding boxes indicate ground-truth defects that YOLOv12 failed to detect but were successfully identified by CrackNet-Weather.

As can be seen, CrackNet-Weather is able to accurately detect subtle, low-contrast, or partially occluded defects that are missed by YOLOv12, particularly under severe rain and snow. This qualitative comparison highlights the superior robustness and discriminative power of CrackNet-Weather for challenging real-world environments.

## 6. Discussion

In addition to the overall quantitative results, we further analyze representative error cases to better understand the model’s limitations under adverse weather conditions. Figure 5 presents three typical examples: class confusion, false positives, and missed detections.

**Figure 8:**
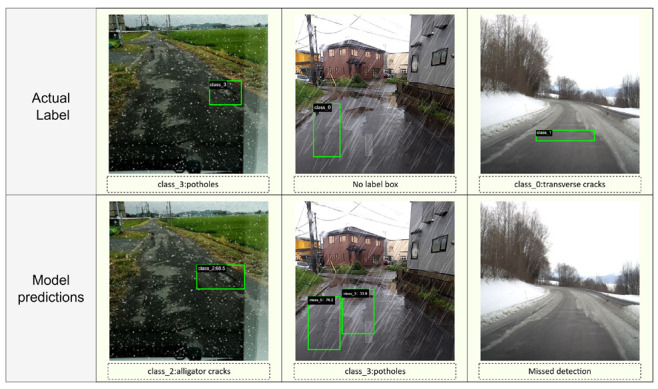
Representative misclassification cases of CrackNet-Weather under adverse weather conditions. The first row shows the ground-truth labels for three scenes, while the second row shows the model’s predictions. (**Left**) Class confusion: a pothole is misclassified as an alligator crack. (**Middle**) False positive: water accumulation is incorrectly detected as a pothole. (**Right**) Missed detection: a longitudinal crack is present in the ground truth but not detected by the model.

As shown in the left column of Figure 8, the model sometimes misclassifies potholes (class_3) as alligator cracks (class_2). This confusion is particularly common when potholes are partially filled with water, snow, or debris, which can alter their boundary shapes and visual textures. In such cases, the edge of the pothole becomes fragmented or network-like, resembling the patterns of alligator cracks. Adverse weather further exacerbates this issue by blurring boundaries and reducing feature distinctiveness. This phenomenon suggests that incorporating more discriminative features or additional context cues may help mitigate class confusion in future work.

The middle column of Figure 8 presents a typical false positive case. In the ground truth, only a single longitudinal crack is annotated. However, the model incorrectly detects an additional defect, identifying a region of water accumulation on the road surface as a pothole (class_3). This type of misclassification frequently occurs under rainy conditions, where surface water can form reflective or irregular patterns that differ in appearance from the surrounding pavement. Factors such as light refraction, surface roughness, and varying water depth can create localized dark or distorted regions that visually resemble the typical signatures of potholes. To mitigate this issue, future work could incorporate multi-frame temporal cues, spectral information, or additional sensor modalities (such as polarization imaging or LiDAR) to more reliably differentiate between water accumulation and real structural defects.

The right column of Figure 8 shows a missed detection, where an obvious longitudinal crack (class_0) is present in the ground truth but not detected by the model. Missed detections often occur in scenes with extremely low contrast or severe occlusion due to snow cover or wet road surfaces. In these conditions, the intensity difference between cracks and the surrounding pavement is diminished, and crack boundaries are partially hidden, making it challenging for the model to extract reliable features. Future efforts could explore multi-modal sensing (e.g., combining RGB with infrared or LiDAR data) or improved attention mechanisms to enhance the detection of subtle or occluded cracks.

Despite these promising results, CrackNet-Weather still exhibits certain limitations and areas for further improvement, as evidenced by the representative misclassification cases analyzed above. We refer to the following limitations specifically:

1. While the model shows robust performance overall, misclassification and missed detection still occur in challenging scenarios, particularly when environmental artifacts such as water accumulation or snow cover visually resemble true pavement defects, or when cracks are faint and difficult to distinguish under low-contrast or occluded conditions. These issues highlight the need for more advanced feature extraction, context modeling, and possibly the integration of multi-modal sensor information (e.g., infrared or LiDAR) to better distinguish between real defects and environmental noise.

2. The current training dataset is limited in scale and diversity. Future efforts should focus on further enriching the dataset to cover a wider range of regions, pavement types, and weather conditions, thereby improving the generalization and adaptability of the model.

3. Although CrackNet-Weather maintains a balance between detection accuracy and computational efficiency, there is still potential for further optimization in model size and inference speed. Approaches such as network pruning, quantization, and lightweight architecture design could facilitate real-time deployment on edge and mobile devices.

4. Another promising future direction is to incorporate semantic segmentation methods after accurate crack detection in order to isolate crack pixels and estimate crack widths. This would enable the identification of fine and narrow cracks that are difficult to capture with detection-based methods alone. Such an extension could greatly benefit the early detection of pavement defects and provide more detailed structural information for preventive road maintenance.

5. The current study relies solely on RGB imagery, which limits the ability to distinguish visually similar artefacts (e.g., water accumulation versus potholes) and to detect faint cracks under low-contrast conditions. Incorporating additional sensing modalities such as thermal infrared or depth could provide complementary cues, which represents an important direction for future work.

6. This study does not include inference speed or latency tests on embedded/edge hardware platforms (e.g., NVIDIA Jetson). While the proposed method achieves favorable complexity–accuracy trade-offs in simulation, its deployment performance on resource-constrained devices remains to be systematically evaluated in future work.

In summary, CrackNet-Weather offers an innovative and practical solution for pavement crack detection under adverse weather and complex pavement conditions. Nevertheless, ongoing optimization in model generalization, adaptability to extreme environments, and engineering deployment will be necessary to fully realize its potential in real-world applications. With continued advances in dataset diversity, model architecture, and multi-modal fusion, the proposed framework holds significant promise for large-scale intelligent road inspection, infrastructure health monitoring, and smart city development.

## 7. Conclusions

In this paper, we proposed CrackNet-Weather, which is a robust and efficient pavement crack detection framework specifically designed to address the challenges posed by adverse weather conditions such as rain and snow. By systematically integrating the Haar Wavelet Downsampling Block (HWDB), Strip Pooling Bottleneck Block (SPBB), and Dynamic Sampling Upsampling Block (DSUB) into the YOLOv12 backbone, the proposed method effectively enhances the extraction and fusion of frequency, contextual, and structural information, significantly improving the detection accuracy of low-contrast, fine, and complex cracks.

Extensive experiments conducted on a diverse and challenging dataset—including both clear and weather-augmented images as well as a cross-domain test on the Norway dataset—demonstrate that CrackNet-Weather outperforms mainstream baseline models. The proposed framework not only achieves a higher mean Average Precision for all major defect categories but also maintains a favorable balance between detection accuracy, model size, and computational complexity. These results validate the effectiveness and practicality of CrackNet-Weather for real-world road inspection and large-scale deployment.

Despite these achievements, several issues remain for future research. In particular, expanding the dataset to cover more geographic regions and additional weather scenarios, as well as further optimizing the model for lightweight deployment, will be critical to improving generalization and adaptability. Incorporating multi-modal sensor information and exploring advanced feature enhancement strategies are also promising directions to further address challenging cases of misclassification and missed detection.

Overall, CrackNet-Weather provides an effective and scalable solution for pavement crack detection under adverse weather, holding substantial potential for application in intelligent transportation infrastructure monitoring and automated road maintenance systems.

## Figures and Tables

**Table 1:** Statistics of the two datasets used in this study, including the number of training, validation, and test sets in each dataset and the number of annotated bounding boxes for each defect class.

Datasets	Total	Train	Val	Class + boxNum			
				Longitudinal Cracks	Transverse Cracks	Alligator Cracks	Potholes
Rain/Snow	2600	2000	600	1307	1288	2098	733
Norway	2914	2039	875	8570	1730	468	461

**Table 2:** Ablation study of each module under rainy and snowy conditions.

Models	SPBB	HWDB	DSUB	Longitudinal		Transverse		Alligator		Potholes		All	
				Rain	Snow	Rain	Snow	Rain	Snow	Rain	Snow	Rain	Snow
YOLOv12-s				13.41	13.13	8.62	7.54	26.42	25.12	15.74	14.81	16.02	15.16
	✓			14.13	13.22	8.83	8.24	28.83	27.72	16.15	15.17	17.02	16.34
		✓		14.31	13.25	8.33	7.02	27.75	28.57	16.14	15.45	17.33	16.24
			✓	13.43	13.07	10.12	7.31	28.75	27.42	15.36	15.33	16.94	15.91
	✓	✓		14.53	13.25	9.26	8.53	29.12	28.77	16.54	15.79	17.63	16.51
	✓		✓	14.42	13.27	9.93	8.17	28.83	28.87	16.82	15.86	17.54	16.32
		✓	✓	14.51	13.32	9.63	8.12	29.24	27.85	16.22	15.43	17.28	16.21
	✓	✓	✓	14.85	13.32	10.24	8.76	29.53	29.43	16.84	15.92	17.88	16.85

**Table 3:** Ablation study of computational complexity and inference speed for each module.

Models	SPBB	HWDB	DSUB	FLOPs	Params	Inference (ms/img)	FPS
YOLOv12-s				21.5	9.25	2.8	357
	✓			29.5	9.10	4.2	238
		✓		18.9	8.19	4.8	208
			✓	21.3	9.25	4.6	217
	✓	✓		26.9	8.03	4.6	217
	✓		✓	29.6	9.12	4.6	217
		✓	✓	18.9	8.21	4.8	208
	✓	✓	✓	26.9	8.05	4.6	217

**Table 4:** Comparison with other mainstream methods under rainy and snowy conditions.

Models	Flops/G	Parameters/M	Longitudinal		Transverse		Alligator		Potholes		All	
			Rain	Snow	Rain	Snow	Rain	Snow	Rain	Snow	Rain	Snow
YOLOv8-s	14.3	11.1	14.61	12.42	8.12	7.74	25.42	25.36	19.25	19.12	16.91	16.06
YOLOv10-s [13]	24.8	8.07	12.41	11.42	7.33	6.62	24.92	24.13	14.75	14.28	14.81	14.14
YOLOv11-s [14]	10.8	9.46	12.12	13.33	8.21	7.06	27.67	26.55	17.22	14.74	16.31	15.46
YOLOv12-s [15]	21.5	9.25	13.41	13.13	7.52	6.34	27.02	26.42	16.04	14.51	16.02	15.16
YOLOv13-s [16]	21.0	9.03	10.22	8.63	6.24	5.95	28.31	26.64	14.42	14.13	14.86	12.61
Ours	26.9	8.05	15.35	13.92	11.44	8.56	29.73	29.33	19.14	17.02	17.88	16.85

**Table 5:** Performance comparison on the Norway pavement defect dataset.

Model	AP_50:95_	AP_50_	Flops/G	Parameters/M
YOLOv8-s	8.13	20.13	14.27	11.14
YOLOv11-s	6.13	16.34	10.81	9.46
YOLOv12-s	7.39	18.82	21.51	9.25
YOLOv13-s	7.52	19.35	21.02	8.05
Faster-rcnn-r18	9.61	26.36	151.31	28.30
Cascade	11.02	26.71	231.00	69.16
CrackNet	9.22	24.24	26.91	8.05

**Table 6:** Model performance under normal and extreme weather conditions.

Model	Normal Weather Dataset		Extreme Weather Dataset	
	AP_50–95_	AP_50_	AP_50–95_	AP_50_
YOLOv12	18.01	40.42	15.16	34.32
CrackNet	19.23	43.25	16.85	38.62

**Table 7:** CrackNet performance under different degradation conditions.

Model	Condition	AP_50–95_	AP_50_
CrackNet	Normal (Rain)	17.88	39.12
	Normal (Snow)	16.85	38.62
	Light Rain	18.40	39.80
	Heavy Rain	15.73	35.21
	Light Snow	17.30	39.20
	Heavy Snow	14.32	33.99
	Gaussian Blur (radius = 1)	16.84	38.09
	Gaussian Blur (radius = 1.5)	16.15	36.93
	Gaussian Blur (radius = 2)	15.28	34.98
	Brightness (factor = 0.6)	14.93	34.21
	Brightness (factor = 1.0)	17.88	39.12
	Brightness (factor = 1.4)	17.02	38.48

**Table 8:** Severity-wise summary on RDD2022-Japan (AP_50:95_). Retention $=\frac{AP_{\mathrm{Heavy}}}{AP_{\mathrm{Light}}}\times 100\%$.

Factor	${\mathrm{AP}}_{\mathrm{Light}}$	${\mathrm{AP}}_{\mathrm{Heavy}}$	Retention (%)	Normalized Drop (%)	Slope (pp/Level)
Rain	18.40	15.73	85.5	14.51	−1.335
Snow	17.30	14.32	82.8	17.23	−1.490
Blur	16.84	15.28	90.7	9.26	−0.780
Brightness ^†^	17.02	14.93	87.7	12.29	−1.045

^†^ Brightness severities ordered as *over-exposed (Light)* → *normal (Normal)* → *under-exposed (Heavy)*; Normal value is 17.88 for reference.

## Data Availability

The dataset used in this study, RDD2022, is publicly available at the following GitHub repository: https://github.com/sekilab/RoadDamageDetector (accessed on 1 June 2025).
